# Importance of Interior Design: An Environmental Mediator for Perceiving Life Satisfaction and Financial Stress

**DOI:** 10.3390/ijerph181910195

**Published:** 2021-09-28

**Authors:** Jeongah Kim, Wookjae Heo

**Affiliations:** 1 College of Engineering, Keimyung University, Daegu 42601, Korea; design1@kmu.ac.kr; 2 School of Hospitality & Tourism Management, Purdue University, West Lafayette, IN 47907, USA

**Keywords:** stimuli–organism–response framework, interior design, mental responses, emotional perception, HLM, SEM

## Abstract

Based on the stimuli–organism–response framework, this study investigates how artistic stimuli (i.e., interior design) influence a person’s mental responses (i.e., situational satisfaction and stress). Prior to checking the main analysis, demographic features were checked to determine whether they were significant precedents to the stimuli by using hierarchical linear modeling. As the main model, structural equation modeling was used to find (a) how stimuli (i.e., interior design) were associated with organisms (i.e., emotional perception) and (b) how organisms were associated with mental responses. The results showed that demographic features were not significantly associated with the stimuli. Stimuli were partially and significantly associated with organisms and the organisms were partially and significantly associated with the mental responses. The study has implications for practitioners in commercial fields who might recognize the importance of interior design and employ their utilities in practical applications.

## 1. Introduction

As is well known, the main purpose of commercial places is to generate profit. However, some commercial places, such as shopping malls, entertaining places (e.g., theaters), hospitality (e.g., hotels), counseling offices and therapeutic stores, help people to be mentally restored and healed (e.g., [1,2,3]). This implies that the environments of commercial places are not isolated from mental health. Rather, those places are associated with daily life and may be related to daily mental status. Even more, the frequency of visits to those sites might be more than the frequency of visits to actual medical facilities, such as hospital and therapists. Therefore, the environments of those facilities are assumed to be important to the mental status of the general population. As such, based on the importance of the environment in commercial places and their daily accessibility, this study investigates the effects of the environment on the mental responses to a commercial place, specifically, a store.

Based on the stimuli–organism–response (SOR) framework [4], the main purpose of this study was to check the effect of environmental stimuli on mental response. Because environmental stimuli can be enormously diverse, such as natural, working, local, rural/urban, psychological/emotional and social environments [5], a set of specific stimuli and corresponding responses was selected in this study. This set of various stimuli is considered an environmental load that results in complex responses, as the SOR framework explains [6]. Therefore, this study explored by what kind of complexity the environmental components were compounded and how they were related to a person’s response [7]. It was discovered that it was hard to notice which environmental component influenced a person when there were multiple environments compounded [8]. As a result, any certain environmental component should be specified in a study so that investigators might understand the specific component’s effect on a person [9].

Therefore, in this study, a specific environmental stimulus was selected, artistic interior design at a commercial store. Artistic interior design is normally known to be a part of marketing performance [10,11]. However, it is not just a marketing performance; it elaborates the consumers’ responses as an environmental stimulus [12,13] by provoking emotional and cognitive responses [14,15]. Therefore, the artistic interior design elements were considered as effective sensory stimuli to consumers [16,17]. The sensory stimuli to the consumers were linked to the mental responses and the corresponding mental responses of the artistic interior design included situational satisfaction and stress at the store. In addition, the commercial store is amongst the general environments where external factors (e.g., lighting, music, layout and others) influence a person and the consumer behavior generates from the general responses to the environment [12]. Consumers’ psychological responses to a store (e.g., consumer satisfaction) were considered a noticeable illustration of environmental effect by a store [18,19]. To sum up, the environmental effect at a store (i.e., artistic interior design) on a person (i.e., consumer) can be one of the representative analytic units for investigating how environments affect a person’s mental response and this study utilizes the specification as an analytic unit.

As explained in the Results and Discussion Sections, this study confirms the importance of environmental stimuli on a mental response. Specifically, the study checks how artistic stimuli are associated with market participants’ mental responses. Therefore, the contribution of the study emphasizes the importance of physical environments on mental responses. The finding can be adopted in various research areas that focus on where a person should take some emotional rest (e.g., hospitality, tourism, therapy, etc.). In addition, this study utilizes artistic stimuli as the specific stimuli. The results show that artistic stimuli are associated with mental response. This finding can be adopted by diverse practitioners who could improve their facilities with art for better mental responses of market participants.

## 2. Theoretical Background and Literature Review

### 2.1. Stimuli–Organism–Response Framework

The SOR framework was introduced by Mehrabian and Russell [4] to understand how internal environments at a store influence a person [9]. The term of internal environments at a store can be considered as interior design [20]. Based on the SOR framework, the internal environments (e.g., interior design) are assumed to influence the primary emotional responses, called an organism in the framework [4]. By following the connection between internal environments (stimuli) and emotional responses (organism), the emotional responses are assumed to affect the behavioral responses (response). The behavioral responses include the verbal or non-verbal communication of preference of a person [4].

The first part of the SOR framework is the environmental effect on a person at a store. Internal environments at a store, such as interior design, are expected to evoke a person’s response. However, as explained in the introduction, a high-load environment may cause unpredictable responses from a person inside the store because a set of various stimuli might produce complex responses [6]. Therefore, a specific environmental component (i.e., artistic interior design) is examined, in this study, to learn how the artistic interior design influences a person’s response at a store. The details of the interior design are explained in Section 2.3.

The second part of the framework is the organism, that is, the emotional responses of a person [4,9]. Generally, the emotional responses are not only one-directional emotions. Rather, the emotional responses can be observed to display various facets, such as intensity, pleasure, discomfort, activation and other multiple forms of emotions [21,22]. Therefore, in this study, multiple emotional responses were measured for the varieties of emotions: good, curious, excited, aroused, focused, annoyed, depressed, bored, nervous and timid. They were categorized into two major emotions, positive emotional perception and negative emotional perception at a store.

Finally, the response is how a person reacts to the environment (i.e., stimuli). Originally, Mehrabian and Russell [4] suggested two reactions, avoidance or approach. This means that a person who experienced the store would show one of two reactions: tendency not to come next time or tendency to come again next time. However, it was too generalized to just have two aspects of behavior. Therefore, Donovan and Rossiter [6] specified the reactions into three categories, each with varying degrees: (a) level of willingness to explore the environment, (b) level of willingness to interact with the others at the environment and (c) level of willingness to show satisfaction with the environment. Therefore, situational satisfaction and stress were selected to measure the three categories of responses in this study, which are explained in detail in the measurement section.

### 2.2. Demographics, Life Satisfaction and Interior Design to Responses

Demographic features are a person’s primary characteristics to be measured when studying a person’s situational behavior. Therefore, the responses (i.e., situational satisfaction and stress) through organism (i.e., positive and negative emotional perceptions) are, at times, considered to be associated with demographic features such as age and gender, specifically in terms of artistic situation [23]. In addition, artistic environments can be associated with high culture, exclusivity and luxury [24,25], regardless of the quality of artwork [26]. This implies that the level of education and income might be associated with art perception. Therefore, in this study, demographic features were tested to determine whether they were actual precedents of art perception.

As Lam [12] explained, interior environments at a store can be considered as situational environments of a consumer. Consumer satisfaction is a representative example of an environmental effect that influences a person’s psychological response to a certain place [19]. However, consumer satisfaction at a store is not a continuous satisfaction, such as life satisfaction, but a situational, spontaneous satisfaction. Considering that art is known to be strongly associated with quality of life or life satisfaction [27], the continuous satisfaction (i.e., life satisfaction) should be controlled when the model is conducted. Because this study focuses on the situational satisfaction obtained from a store, the continuous satisfaction level should be checked to determine whether it might influence the art perception. Therefore, in this study, life satisfaction was first checked to determine whether it was an actual precedent to art perception.

In addition, components of the interior design (i.e., stimuli) should be specified to refine the research model. Three components of interior design (i.e., decoration, color and lighting) were selected as the stimuli at a store. First, decoration was considered in this study. As various researchers explained (e.g., [6,8,9]) that art perception and a person’s response are associated with the complexity of environments. Based on Nasar [28], the environmental complexity includes varieties of decorations and visual richness. Second, color is also known to be an important interior design element that is associated with a person’s response, as well as emotional perception [29,30,31]. Specifically, a certain combination of colors has its own meanings [32], so that the colors are amongst the most significant factors at a store [33]. Third, lighting is considered as a potential environmental stimulus to influence consumers at a store [34,35,36]. As a result, three components of interior design (i.e., decoration, color and lighting) were the stimuli at a store in this study.

Finally, by considering that the environments, in this study, were specified as the interior design elements at a store, the responses were specified as consumers’ responses, including situational satisfaction and situational stress toward the store. In terms of situational satisfaction, as Donovan and Rossiter [6] suggested, one of the responses, as indicated in the SOR framework, is the level of willingness to show satisfaction about the environment (i.e., artistic interior design elements). Therefore, studying the respondents’ situational satisfaction was a possible way to measure the mental responses.

In the case of situational stress, financial stress was used in this study. Bearing in mind that the commercial environment (i.e., store) was given to consumers in this study, consumers were unsurprisingly exposed to the utility-seeking situation. It implies that monetary concern, which is a type of stress [37], may be inevitable in a person’s responses. Specifically, financial stress is a combination of a stressful situation and stress response [38,39]; this means that stress occurs where there is stressful situation and a stressful environment [40]. In addition, Sapolsky [41] explained that stress response (i.e., physiological response) is observed as being situational. For instance, financial stress was reported to be associated with situational satisfaction, such as that at the workplace [42]. As a result, the financial stress scale was developed based on the fact that a person perceives uncertainty as a potential risk or cause of harm [38]. Within the financial stress responses, Heo et al. [38] explained that there are multiple types of responses, such as the affective response and interactive responses. These responses are also conceptually connected to the SOR responses explained by Donovan and Rossiter [6]. The level of willingness to explore is associated with the affective response, while the willingness to interact with others is conceptually associated with the interactive responses.

### 2.3. Conceptual Framework and Research Hypotheses

**Figure 1:**
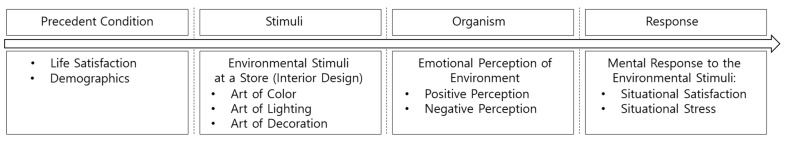
Conceptual model of the study.

Based on the theoretical background (i.e., SOR framework) and literature, the conceptual framework is shown in Figure 1. In this study, there was a precedent added to the SOR framework. Because the general condition of an individual, such as life satisfaction and demographic factors, can be the precedents of the SOR model, they were added to the model. In addition, because the environment was the artistic interior design at a store, in this study, mental responses were measured by following the specific situation. As explained above, mental responses in this study include (a) situational satisfaction (i.e., satisfaction of a store) and (b) situational stress (i.e., financial stress).

Prior to checking the above conceptual framework, the first and second research hypotheses were intended to check whether demographic factors and life satisfaction influence the perception of artistic environment in a certain situation (i.e., interior design at a store).

**Hypothesis** **1** **(H1).** *Demographic features are associated with the perception of art of interior design.*

**Hypothesis** **2** **(H2).** *Life satisfaction is associated with the perception of art of interior design. Based on the results from the first two hypotheses, the following hypotheses checked the conceptual model as shown in Figure 1.*

**Hypothesis** **3** **(H3).** *Interior design elements (i.e., art of color, art of lighting and art of decoration) are associated with positive/negative emotional perceptions of the environment.*

**Hypothesis** **4** **(H4).** *Positive/negative emotional perceptions of the environment are associated with situational responses (i.e., situational satisfaction, affective financial stress and interpersonal financial stress).*

## 3. Data and Methodology

### 3.1. Data

The sample was collected in South Korea and a random sampling survey was employed. An online survey agency was employed to perform random sampling. The survey company in South Korea randomly sent survey invitation emails to those who were listed in the company’s contact list. When a recipient of the invitation email accepted the invitation, the person could participate in the survey. Therefore, the study employed random selection with voluntary willingness to participate.

The sample size was 691, with 344 (49.78%) females and 347 (50.22%) males. The average age of the total sample was 39.29, with a standard deviation of 10.83. The minimum age requirement to answer the survey was 20 and the oldest age was 59. Working age was employed in this study. All respondents were given a situation in which they were shopping at a store. All respondents saw pictures of the same store with two different kinds of interior design, because one interior design did not secure the diversity of situational effects. Among 691 samples, 347 samples were randomly selected to see the Type A interior design. The other 344 samples were shown the Type B interior design. Because of copyright issues, the pictures were only allowed to be used in a survey; the pictures cannot be shown in the manuscript. However, by using two kinds of interior design of one store, the study secured the diversity of effects produced by interior design, as well as excluding the exogenous effects (e.g., external effects by differences in places). In other words, the usage of two different kinds of interior design had the purpose to exclude a nonsystematic error that could have occurred by using only one specific type of interior design. For instance, if only one specific type of interior design had been surveyed in the research, then consumers might have been biased by the picture itself. Therefore, two different types of pictures were shown in the survey as a strategy for the survey method.

In terms of educational level of the respondents, 121 respondents had completed high school or lower; 494 respondents had completed an associate degree (AA) or bachelor’s degree (BA) from a college or equivalent educational institute; 76 respondents had a graduate degree or higher-level education, such as professional certificate and doctoral degree. Among 691 samples, 360 respondents lived with a significant partner or were married; the other 331 were single, including unmarried, divorced and widowed. The monthly income of respondents was as follows: 72 earned lower than KRW 2 million; 240 respondents earned between KRW 2 million and KRW 4 million; 174 respondents earned from KRW 4 million to KRW 6 million; 124 respondents reported their income was between KRW 6 million and KRW 8 million; 81 participants answered that their income was over KRW 8 million.

### 3.2. Analytics

For the first two hypotheses (H1 and H2), hierarchical linear modeling (HLM) was implemented. By adding additional factors in multiple linear models, HLM helps to find the effective factors on an outcome [43]. In this study, the nested model was utilized. The nested model utilizes the blocks for each set of factors to test the significance of a set of predictors [44]. By employing HLM, the two functions (Functions (1) and (2)) below were employed in this study.

(1) $$Y_{i}=a_{i}+\sum^{\;}\left(b_{i}x_{i}\right)+e_{i}$$

(2) $$Y_{j}=a_{j}+b_{LS}LS+\sum^{\;}\left(b_{j}x_{j}\right)+e_{j}$$

where *Y* denotes the art perception of interior design to sum three interior design elements (i.e., art of color, art of lighting and art of decoration), which are explained in the measurement section, below; *i* is the model without life satisfaction; *j* is the model with life satisfaction as an additional predictor; *LS* denotes life satisfaction; *b* and *s* are coefficients; *e* and *s* are error terms.

In terms of the other hypotheses (H3 and H4), structural equation modeling (SEM) was utilized for checking the association among stimuli, organism and response, as shown in Figure 1 above. SEM is well known for checking the directional association among selected variables by using the latent concept [45,46]. The construct of SEM follows the conceptual model of this study (see Figure 1). To implement the two methodologies above (i.e., HLM and SEM), Stata 15.1 was utilized.

### 3.3. Measurement

Demographic features were measured by asking the respondents for their gender, age, educational level, marital status and income level. For gender categorization, respondents were asked to answer male or female. Age was measured with the actual age of the respondent. For education level categorization, respondents were asked to answer by choosing one of three options: graduate high school; college degree, including associate degree and bachelor’s degree; higher than graduate degree. In the analytic stage (i.e., HLM), the education level was utilized by recoding as dummy variables. For marital status categorization, respondents were asked to answer single or married. Finally, income level was measured with monthly income by choosing from the following levels: lower than KRW 2 million, between KRW 2 million and KRW 4 million, between KRW 4 million and KRW 6 million, between KRW 6 million and KRW 8 million, and over KRW 8 million. KRW (won) is the Korean currency; KRW 1100 is equivalent to approximately USD 1.

Life satisfaction was measured with the five items introduced by [47]. After introducing it by referencing Diener et al. [47], the validity of the satisfaction-with-life scale (SWLS) was confirmed multiple times (e.g., [48,49,50,51]). The scale included five items: (a) “In most ways my life is close to my ideal”; (b) “The conditions of my life are excellent”; (c) “I am satisfied with my life”; (d) “So far I have gotten the important things I want in life”; (e) “If I could live my life over, I would change almost nothing”. Responses to all five items were answered using a 7-point Likert scale from 1 (strongly disagree) to 7 (strongly agree). The total sum of the answers was used for indicating the respondent’s life satisfaction. Therefore, a higher number indicated a higher life satisfaction. In this study, with 691 samples, the Cronbach’s alpha of the SWLS was 0.87, which was sufficiently reliable over 0.70 [52].

To measure the environmental stimuli, emotional perception and mental response at a store (the store of a Korean fashion brand (i.e., KUHO) was used for this study), six pictures were shown to the respondents. All six pictures showed one store’s interior design from various angles, but there were two different sets of interior design. Each set of interior design consisted of three pictures based on the same interior design. All respondents were randomly shown one of two sets. By showing two sets of interior design of the same store, the perception of art was intended to be differentiated and the exogenous effect excluded.

In terms of environmental stimuli, three questions were asked regarding artistic stimuli from the interior design. Specifically, art of color, art of lighting and art of decoration were investigated by asking the respondents to rate the following three statements: (a) “I can feel the artistic expression by seeing the color of the store”; (b) “I can feel the artistic expression by seeing the lighting of the store”; (c) “I can feel the artistic expression by seeing the decorations of the store”. All statements were asked to be rated on a 5-point scale from 1 (strongly disagree) to 5 (strongly agree). Each statement was utilized for art of color, art of lighting and art of decoration, respectively, as shown in Figure 1. A higher number meant that a respondent felt a stronger response to artistic expression in color, lighting and decoration.

Emotional perception in a store was measured with ten items. Five items were used for measuring positive emotional perception and the other five items were utilized for measuring negative emotional perception. The questionnaire for measuring emotional perception of a store was adapted from Vukadin, Lemoine and Badot [53]. The first five items to measure positive emotional perception were as follows: “At the current store, do you feel (a) good; (b) curious; (c) excited; (d) aroused; and (e) focused?” The five items to measure negative emotional perception were as follows: “At the current store, do you feel (a) annoyed; (b) depressed; (c) bored; (d) nervous; and (e) timid?” Total sums of the answers were used for indicating the respondent’s positive and negative emotional perception. Therefore, a higher number of positive perceptions indicated that a respondent perceived the art at a store more emotionally positively, while a higher number of negative perceptions indicated that a respondent perceived the art at a store more emotionally negatively. In this study, reliability of these two emotional perceptions were confirmed, with the Cronbach’s alphas being 0.82 and 0.78, respectively, for positive and negative emotional perception.

Three facets of mental responses were measured: (a) situational satisfaction of a store, (b) situational stress by seeing affective financial stress and (c) situational stress by seeing interpersonal financial stress. As explained above, three kinds of levels can be measured for understanding a person’s mental responses: (a) level of willingness to show satisfaction from the environment, (b) level of willingness to explore the environment and (c) level of willingness to interact with the others in the environment. Considering that the given situation was a store, in this study, it was possible to measure the following: (a) the level of willingness to be satisfied with the store (i.e., situational satisfaction); (b) the level of willingness to explore the store can measure how a person was psychologically and whether they were stressed at the store (i.e., affective financial stress); (c) the level of willingness to interact with others at the store (i.e., interpersonal financial stress).

First, situational satisfaction was measured with a question: “Are you satisfied with the current store?” The question was answered on a 5-point scale from 1 (strongly disagree) to 5 (strongly agree). Therefore, a higher score indicated higher satisfaction. Second, items relating situational stress, including affective financial stress and interpersonal financial stress, were adapted from Heo et al. [38]. Heo et al. [38] introduced a comprehensive financial stress scale (APR financial stress scale) using multiple dimensions, such as affective reaction, interpersonal response and physiological responses. Because this study showed pictures, physiological responses were not a valid measure of financial stress. Therefore, two dimensions (i.e., affective reaction and interpersonal responses) were employed in this study. Even if the APR financial stress scale has been relatively recently introduced, there are multiple literature sources to check the reliability and validity for its use in research (e.g., [39,54,55]). The three items for measuring affective financial stress were as follows: (a) “I feel sad because of my financial situation”; (b) “I feel anxious because of my financial situation”; (c) “I am easily irritated because of my financial situation”. The three items for measuring interpersonal financial stress were as follows: (a) “My financial situation interferes with my daily job performance”; (b) “I often argue with my spouse/significant other because of financial matters”; (c) “I frequently avoid attending family events because of my financial situation”. In this study, reliability of these two types of financial stress were confirmed, with the Cronbach’s alphas being 0.94 and 0.84, respectively, for affective financial stress and interpersonal financial stress.

## 4. Results

### 4.1. Descriptive Summary

As shown in Table 1, the average life satisfaction of the sample was 19.46 (SD = 5.86). The average for situational satisfaction was 3.50 (SD = 0.90). Simply comparing the two types of satisfaction with a variance comparison, they were significantly different (*f* = 42.39, *p* < 0.001); life satisfaction was significantly higher than situational satisfaction. This implies that satisfaction can differ depending on the type and the situation, such as environments. The perception of the environment was measured by analyzing three aspects: color, lighting and decoration. The averages were 3.41 (SD = 0.99), 3.13 (SD = 1.01) and 3.37 (SD = 0.99), respectively, for interior design’s color, lighting and decoration. The average score of negative emotional perception at a store was lower than the score of positive perception. Positive emotional perception at a store was 15.09 (SD = 3.41), but negative perception was 10.25 (SD = 3.10). Finally, regarding financial situational stress, the scores were 8.08 (SD = 3.49) and 5.96 (SD = 2.69), respectively, for affective stress and interpersonal stress.

### 4.2. Hierarchical Linear Modeling: H1 and H2

Table 2 shows the answers to the first and second hypotheses (H1 and H2). First, none of the demographic factors were significantly associated with the output, which was artistic perception of interior design (i.e., sum of color, lighting and decoration). In addition, the *F* statistic was not significant at the level of 0.05. This means that the model was not well fitted in terms of demographic factors. Therefore, to answer the first hypothesis (H1), demographic factors were not a precedent to the environmental stimuli. No specific demographic factor influenced the perception of art at a store, which, paradoxically, implied that an artistic environment might not be biased by the surroundings.

On the other hand, as shown in the last two columns of Table 2, life satisfaction was a significant precedent (*b* = 0.06, *p* < 0.01) to artistic perception of the interior design at a store. The *R*^2^ was increased from 0.02 to 0.04 when considering the factor of life satisfaction. In addition, the model became significant (*F* = 2.65, *p* < 0.01); therefore, the model fit was good. The statistic for the block (*F* = 11.26, *p* < 0.001) was significant as well, which implied that adding life satisfaction improved the model significantly. As a result, the answer to the second hypothesis (H2) was that life satisfaction should be a precedent for artistic perception of interior design (i.e., perception of environmental stimuli). This implies that life satisfaction is strongly associated with the perception of beneficial surroundings. To sum up the HLM results, demographic factors can be excluded from the conceptual model (see Figure 1), but life satisfaction should be kept in the conceptual model.

### 4.3. Structural Equation Modeling: H3 and H4

Based on the results from the first and second hypotheses, SEM tested how well the conceptual model fit among interior design (i.e., color, lighting and decoration), emotional perception (positive and negative) and situational mental responses (i.e., satisfaction and financial stress). The model fit of SEM was as follows: root mean square error of approximation (RMSEA) = 0.082; Akaike information criterion (AIC) = 27,152.824; Bayesian information criterion (BIC) = 27,416.036; comparative fit index (CFI) = 0.960; Tucker Lewis index (TLI) = 0.929. In terms of RMSEA, values lower than 0.06 were preferred [56]; however, a value of about 0.08 was still acceptable [57]. CFI and TLI were recommended to be higher than 0.90 [45,46]. Therefore, the empirical model found in this study showed good/acceptable goodness-of-fit. This means that the dataset used in this study matched the conceptual model well.

**Figure 2:**
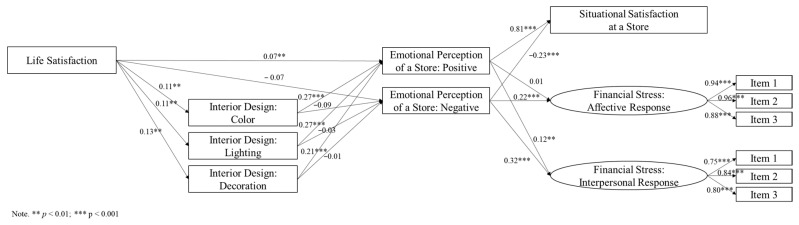
Structural equation modeling results.

As shown in Figure 2 and Table 3, the same result from the second hypothesis was confirmed. Life satisfaction was significantly associated with the perception of art (i.e., art of color, art of lighting and art of decoration), given as environmental stimulus in this study. Life satisfaction was positively associated with art of color (*b* = 0.11, *p* < 0.01), art of lighting (*b* = 0.11, *p* < 0.01) and art of decoration (*b* = 0.13, *p* < 0.01). This means that those who had higher life satisfaction would have a higher level of perceiving art from holistic interior design through all the detailed aspects of interior design, such as color, lighting and decoration. Interestingly, life satisfaction was associated with only positive emotional perception (*b* = 0.07, *p* < 0.05), but not associated with negative emotional perception (*b* = −0.07, *p* = 0.063).

Figure 2 and Table 3 show the answers to the third and fourth hypotheses (H3 and H4). First, three interior design elements were all significantly and positively associated with positive emotional perception (H3). Art of color was significantly and positively associated with positive emotional perception (*b* = 0.27, *p* < 0.001); art of lighting was significantly and positively associated with positive emotional perception (*b* = 0.27, *p* < 0.001); art of decoration was significantly and positively associated with positive emotional perception (*b* = 0.21, *p* < 0.001). This implies that those who perceive arts of interior design (i.e., color, lighting and decoration) are more likely to feel positive emotions (i.e., good, curious, excited, aroused and focused) from a store. However, in terms of negative emotional perception, the third hypothesis (H3) was partially rejected. Art of color was not significantly associated with negative emotional perception (*b* = −0.09, *p* = 0.066); art of lighting was not significantly associated with negative emotional perception (*b* = 0.03, *p* = 0.566); art of decoration was not significantly associated with negative emotional perception (*b* = −0.01, *p* = 0.785). This implies that interior design was not associated with feeling negative emotions (i.e., annoyed, depressed, bored, nervous and timid) at a store. As a result, the third hypothesis (H3), that is, interior design elements, as environmental stimuli, influence the emotional perception as an organism (i.e., positive and negative emotional perception), was partially accepted.

Second, emotional perceptions were all significantly associated with situational satisfaction (H4). Positive emotional perception was significantly and positively associated with situational satisfaction (*b* = 0.81, *p* < 0.001); negative emotional perception was significantly and negatively associated with situational satisfaction (*b* = −0.23, *p* < 0.001). Those who had positive emotional perception of a store were more likely to show a higher level of situational satisfaction. However, those who had negative emotional perception of a store tended to have a lower level of situational satisfaction.

In terms of situational financial stress as part of the mental response, emotional perceptions were partially associated with situational stress (H4). Positive emotional perception was not associated with affective financial stress (*b* = 0.01, *p* = 0.789) but significantly associated with interpersonal financial stress (*b* = 0.12, *p* < 0.01). Those who received a positive feeling from a store did not report situational financial stress but showed the situational interpersonal financial stress. However, negative emotional perception was significantly associated with financial stress (i.e., affective financial stress and interpersonal financial stress). Negative emotional perception was significantly associated with affective financial stress (*b* = 0.22, *p* < 0.001) and with interpersonal financial stress (*b* = 0.32, *p* < 0.001). Those who received a negative feeling from a store reported a higher level of situational financial stress.

The findings of six sub-hypotheses reported further results, as shown in Table 4. These included multiple mediation effects: (a) mediating effect of interior design (environmental stimuli) between life satisfaction (precedent) and emotional perception of a store (organism) and (b) mediating effect of emotional perception (organism) between interior design (environmental stimuli) and situational satisfaction and stress (mental responses). Consequently, the mediating effects found by six sub-hypotheses confirmed the conceptual model introduced above (see Figure 1).

As shown in Table 4, art of color mediated between life satisfaction and positive emotional perception with an indirect effect of 0.03 (=0.11 × 0.27). This means that color, as an interior design element, as well as an environmental stimulus, amplified the positivity in the organism. Similar to the art of color, the art of lighting and the art of decoration mediated between life satisfaction and positive emotional perception with an indirect effect of 0.03 (=0.11 × 0.27) and 0.03 (=0.13 × 0.21), respectively. This means that lighting and decoration as interior design elements, as well as environmental stimuli, amplified the positivity in the organism.

In addition, positive emotional perception mediated between interior design (environmental stimuli) and situational satisfaction (mental responses): (a) between art of color and situational satisfaction with an indirect effect of 0.22 (=0.27 × 0.81); (b) between art of lighting and situational satisfaction with an indirect effect of 0.03 (=0.27 × 0.81); (c) between art of decoration and situational satisfaction with an indirect effect of 0.17 (=0.21 × 0.81). Those who perceived the interior design as an artistic form (i.e., color, lighting and decoration) felt positive emotions (i.e., good, curious, excited, aroused and focused) at a store, leading to situational satisfaction as a mental response. Furthermore, positive emotional perception mediated between interior design (environmental stimuli) and interpersonal financial stress (i.e., situational mental responses): (a) between art of color and interpersonal financial stress with an indirect effect of 0.03 (=0.27 × 0.12); (b) between art of lighting and interpersonal financial stress with an indirect effect of −0.03 (=0.27 × 0.12); (c) between art of decoration and interpersonal financial stress with an indirect effect of 0.03 (= 0.21 × 0.12).

## 5. Discussion, Implications and Limitations

In this study, multiple findings are explained. First, there was not a significant association between demographic features and art perception at a store. Anyone can feel art, regardless of their income level, education level and gender. This result seems to be contrasting with the previous literature, such as Smith and Smith [23]. However, it might depend on what kinds of art are perceived by a person at a store. Specifically, Hagtvedt and Patrick [26] explained that the general connotation of art would be associated with consumers’ perception; this means that different kinds of art influence different kinds of demographic categories. As a result, the finding in this study implies that there were no differences in art perception based on demographic features. There should be different levels of art perception regardless of demographic features, where the artistic environment might not be biased by the surroundings. Therefore, to encourage specific mental responses at a store, art might be useful regardless of demographic features. For practitioners in commercial areas such as hospitality, counseling offices, aesthetic stores and others, the finding has implications that artistic interior design is useful regardless of their clients.

Then, the question moves on to how artistic interior design stimuli influence people at commercial places such as a store. The pathway from life satisfaction to mental responses (i.e., situational satisfaction and stress) in Figure 2 provides the answers. In terms of life satisfaction, those who had higher life satisfaction had a higher level of artistic perception from general interior design through all three components of interior design (i.e., color, lighting and decoration). This means that life satisfaction was a significant precedent to the perception of stimuli (i.e., art perception of interior design). The environmental stimuli, such as the artistic interior design elements at a store, could be better captured by a person when the person has higher life satisfaction. Specifically, life satisfaction and situational satisfaction were associated by only using the mediating effect of interior design (i.e., environmental stimuli) and emotional perception (i.e., organism), as shown in Figure 2 and Table 3 and Table 4. Having higher life satisfaction was positively associated with three interior design elements (art of color, art of lighting and art of decoration), which increased positive emotional perception (i.e., organism). Finally, the increased positive emotional perception led to a higher level of situational satisfaction (i.e., mental responses). Therefore, the conceptual model (Figure 1) was confirmed by the significant mediation of interior design and emotional perception toward situational satisfaction. This provides practical implications to practitioners in commercial areas who help clients’ mental responses. The pathway from interior design (i.e., stimuli) and emotional perception (i.e., organism) to situational satisfaction and stress (i.e., mental responses) emphasizes the importance of color, lighting and decoration of interior design.

Specifically, all three components of art were found to be significant stimuli at a store influencing the organism and mental responses. Three types of art of interior design (i.e., environmental stimuli) were positively associated with positive emotional perception (i.e., organism), which eventually significantly associated with situational satisfaction and interpersonal financial stress (i.e., mental response). Finally, it confirmed the conceptual model that environmental stimuli, organism and mental responses were significantly associated.

However, the paper still has a few limitations because this study employed an academic investigation instead of a large number of practical exercises. First, the survey questionnaires about consumers’ responses were delimited by an academic approach aimed to understand. For instance, the scale of affective financial stress and interpersonal financial stress are the proxy method to check whether the environment caused consumers mental health issues. The usage of the financial stress scale is the best method so far, but, in future research, any actual observation and experiments would allow for a better understanding of the association between financial stress and environmental stimuli. Second, the treatment was given as pictures. Because the efficient way to collect a large-sized sample through the survey method was using pictures in the questionnaire, this study utilized the efficient way. However, similarly to the first limitation, actual observation and experiments in future research are expected to confirm the results of this study.

## 6. Conclusions

The current paper studies the importance of interior design elements, including color, lighting and decoration. Practitioners may ask how to utilize color, lighting and decoration. Those utilizations might be found in the literature. For instance, cool tones at a store might help people to feel restored [30,58,59,60]. Some researchers claim that, by seeing warm color tones at a store, people might feel a higher level of anxiety [61] and show distractions [62]. Therefore, the artistic directors of those places where clients should be mentally restored (e.g., hospitality, counseling offices, aesthetic stores, etc.) might need to understand that a specific color is associated with specific clients’ reactions. In the case of lighting, lighting itself are reported to be associated with emotional perceptions at a store [35,63], similarly to the findings from this study. However, lighting depends on what kinds of restorative facilities are requested at a commercial place. Specifically, brighter light or darker light, compared to surrounding spaces, evokes different interests among people [64,65]. Therefore, practitioners at a restorative commercial place should understand the appropriate balance of lighting. To sum up, the findings from this study provide indications for useful utilization of artistic environments that can be adopted in diverse spaces, such as hospitals, counseling offices and therapeutic practices. Based on the present study’s result that artistic environments are stimuli that influence mental responses, future research would be expected to expand the concept of artistic environments toward comprehensive artistic effects of environments, including performing arts and visual arts. By exploring the comprehensive effects of artistic environments, future research would be expected to be more realistic and practical in professional areas.

## Figures and Tables

**Table 1:** Descriptive summary of major scales (*n* = 691).

	Mean	SD	Min.	Max.
Life satisfaction	19.46	5.86	5	35
Art of color	3.41	0.99	1	5
Art of lighting	3.13	1.01	1	5
Art of decoration	3.37	0.99	1	5
Positive emotional perception	15.09	3.41	5	25
Negative emtional perception	10.25	3.10	5	25
Situational satisfaction	3.50	0.90	1	5
Affective financial stress	8.08	3.49	3	15
Interpersonal financial stress	5.96	2.69	3	15

**Table 2:** Hierarchical linear modeling with nested regression (*n* = 648).

	Demographic		Demographic and LS	
	Coef.	S.E.	Coef.	S.E.
Life satisfaction			0.06 **	0.02
Gender (ref. = male)	0.46	0.19	0.44 *	0.19
Age	0.00	0.01	0.01	0.01
Education (ref. = high school)				
College	−1.51	1.76	−1.72	1.75
Graduate or higher	−1.93	1.78	−2.28	1.77
Marital (ref. = single)	0.14	0.25	0.06	0.25
Income level (ref. = lower than 2 mil.)				
Between 2 million and 4 million	0.17	0.34	0.13	0.33
Between 4 million and 6 million	0.25	0.36	0.17	0.36
Between 6 million and 8 million	0.59	0.38	0.36	0.39
Over 8 million	0.53	0.41	0.25	0.42
Constant	10.81 ***	1.82	9.91 ***	1.83
*R* ^2^	0.02		0.04	
Δ*R*^2^	0.02		0.02	
Model *F*	1.67		2.65 **	
Block *F*	1.67		11.26 ***	

Note: * *p* < 0.05; ** *p* < 0.01; *** *p* < 0.001. Reference group for gender is male; reference group for education is graduate high school; reference group for marital status is single; reference group for income level is lower than KRW 2 million. The currency for income level is KRW (Korean won). LS denotes life satisfaction; Coef. means coefficient; S.E. is standard error.

**Table 3:** Direct effects between factors.

Predictor	Criterion	Coefficient	S.E.	Hypotheses
LS	PEP	0.07 **	0.03	-
LS	NEP	−0.07	0.04	-
LS	CLR	0.11 **	0.04	H2 accepted
LS	ILMN	0.11 **	0.04	H2 accepted
LS	DCR	0.13 **	0.04	H2 accepted
CLR	PEP	0.27 ***	0.04	Part of H3 accepted
CLR	NEP	−0.09	0.05	Part of H3 rejected
LGT	PEP	0.28 ***	0.04	Part of H3 accepted
LGT	NEP	−0.03	0.05	Part of H3 rejected
DCR	PEP	0.21 ***	0.03	Part of H3 accepted
DCR	NEP	−0.01	0.04	Part of H3 rejected
PPE	SS	0.81 ***	0.03	Part of H4 accepted
PEP	AFS	0.01	0.04	Part of H4 rejected
PEP	IFS	0.12 **	0.04	Part of H4 accepted
NEP	SS	−0.23 ***	0.03	Part of H4 accepted
NEP	AFS	0.22 ***	0.04	Part of H4 accepted
NEP	IFS	0.32 ***	0.04	Part of H4 accepted

Note: ** *p* < 0.01; *** *p* < 0.001. S.E. is standard error; LS means life satisfaction; CLR is art of color of interior design; LGT means art of lighting of interior design; DCR denotes art of decoration of interior design; PEP is positive emotional perception; NEP denotes negative emotional perception; SS is situational satisfaction; AFS means affective financial stress; IFS means interpersonal financial stress.

**Table 4:** Indirect effects among significant factors.

Predictor	Mediator	Criterion	Coefficient
LS	CLR	PEP	0.03
LS	LGT	PEP	0.03
LS	DCR	PEP	0.03
CLR	PEP	SS	0.22
CLR	PEP	IFS	0.03
LGT	PEP	SS	0.22
LGT	PEP	IFS	0.03
DCR	PEP	SS	0.17
DCR	PEP	IFS	0.03

Note: S.E. is standard error; LS means life satisfaction; CLR is art of color of interior design; LGT means art of lighting of interior design; DCR denotes art of decoration of interior design; PEP is positive emotional perception; SS is situational satisfaction; IFS means interpersonal financial stress.

## Data Availability

Data sharing is not applicable to this article.
